# Extracellular Vesicles From the Cotton Pathogen *Fusarium oxysporum* f. sp. *vasinfectum* Induce a Phytotoxic Response in Plants

**DOI:** 10.3389/fpls.2019.01610

**Published:** 2020-01-10

**Authors:** Mark R. Bleackley, Monisha Samuel, Donovan Garcia-Ceron, James A. McKenna, Rohan G. T. Lowe, Mohashin Pathan, Kening Zhao, Ching-Seng Ang, Suresh Mathivanan, Marilyn A. Anderson

**Affiliations:** ^1^Department of Biochemistry and Genetics, La Trobe Institute for Molecular Science, La Trobe University, Bundoora, VIC, Australia; ^2^Bio21 Institute, University of Melbourne, Parkville, VIC, Australia

**Keywords:** fungi, extracellular vesicle (EV), host-pathogen interaction, polyketide, pigment

## Abstract

Extracellular vesicles (EVs) represent a system for the coordinated secretion of a variety of molecular cargo including proteins, lipids, nucleic acids, and metabolites. They have an essential role in intercellular communication in multicellular organisms and have more recently been implicated in host-pathogen interactions. Study of the role for EVs in fungal biology has focused on pathogenic yeasts that are major pathogens in humans. In this study we have expanded the investigation of fungal EVs to plant pathogens, specifically the major cotton pathogen *Fusarium oxysporum* f. sp. *vasinfectum*. EVs isolated from *F. oxysporum* f. sp. *vasinfectum* culture medium have a morphology and size distribution similar to EVs from yeasts such as *Candida albicans* and *Cryptococcus neoformans*. A unique feature of the EVs from *F. oxysporum* f. sp. *vasinfectum* is their purple color, which is predicted to arise from a napthoquinone pigment being packaged into the EVs. Proteomic analysis of *F. oxysporum* f. sp. *vasinfectum* EVs revealed that they are enriched in proteins that function in synthesis of polyketides as well as proteases and proteins that function in basic cellular processes. Infiltration of *F. oxysporum* f. sp. *vasinfectum* EVs into the leaves of cotton or *N. benthamiana* plants led to a phytotoxic response. These observations lead to the hypothesis that *F. oxysporum* f. sp. *vasinfectum* EVs are likely to play a crucial role in the infection process.

## Introduction

Extracellular vesicles (EVs) are involved in intercellular communications in all kingdoms of life (Pathirana and Kaparakis-Liaskos, 2016; Maas et al., 2017; Bielska et al., 2018; Rybak and Robatzek, 2019). These small, lipid bilayer encapsulated particles provide a mechanism for the coordinated transport of heterogeneous cargo. This is not limited to transport between cells within an organism but also extends to cross kingdom interactions such as those between microbial pathogens and their hosts (Schorey et al., 2015; Rutter and Innes, 2018). The best characterized EVs are from mammalian cells; their cargo includes nucleic acids, proteins, lipids, and metabolites (Kalra et al., 2012; Maas et al., 2017). Uptake of EVs by target cells induces changes in gene/protein expression that consequently lead to altered physiology and other phenotypes (Greening and Simpson, 2018). Our understanding of EVs at the host-pathogen interface comes largely from the study of outer membrane vesicles (OMVs) produced by Gram negative bacteria that infect humans (Pathirana and Kaparakis-Liaskos, 2016). OMVs contribute to the host–pathogen interaction through their cargo, which can induce immune responses (Ellis et al., 2010), contribute to adhesion to mucosal surfaces (Olofsson et al., 2010), act as virulence factors (Bomberger et al., 2009) or toxins (Horstman and Kuehn, 2000), degrade antibiotics (Kim et al., 2018), acquire trace nutrients (Prados-Rosales et al., 2014), and function in inter-microbe competition (Vasilyeva et al., 2008).

Investigation of fungal EVs is in its relative infancy compared to mammalian EVs or bacterial OMVs with most studies on pathogenic yeasts that infect humans (reviewed in Bleackley et al., 2019). Like mammalian EVs and bacterial OMVs, fungal EV cargo consists of proteins, lipids, and nucleic acids. Polysaccharides are also transported by fungal EVs (Griffith et al., 2004; da Silva et al., 2015). Proteomic characterization of the cargo in EVs from pathogenic yeast has led to predicted roles in stress responses, and metabolism of various cellular building blocks including proteins, lipids, and carbohydrates. Functional analysis of EVs *in vitro* supports several roles for these vesicles in host–pathogen interactions. For example, EVs from a number of pathogenic yeast induce responses when applied to cultured innate immune cells, indicating EVs are probably sensed by the host during infection (Oliveira et al., 2010; Wolf et al., 2015; da Silva et al., 2016). Antifungal drug resistance is also linked to EV production; EVs from biofilms produced by the major human fungal pathogen *Candida albicans* contribute to resistance to antifungal azoles (Zarnowski et al., 2018) and *S. cerevisiae* EVs have a protective function against both caspofungin and antifungal peptides(Zhao et al., 2019). Virulence has also been linked to EV production. Hypervirulence of a *Cryptococcus gattii* strain is facilitated by intra-cellular communication between fungal cells mediated by EVs (Bielska et al., 2018).

Little is known about the role of EVs in plant–fungal interactions, although there has been speculation based on the study of mammalian yeast pathogens (Samuel et al., 2015; Boevink, 2017). Experimental investigation into the role for EVs in the plant–fungal interaction has focused on EVs produced by the plant, defense related cargo, and the effect they have on fungi (Rutter and Innes, 2018). Multivesicular bodies, which are the site of biogenesis for many EVs, accumulate at plasmodesmata during fungal infections leading to a potential role for vesicles in the callose deposition that isolates dying cells from living cells (An et al., 2006; Bohlenius et al., 2010; Li et al., 2018). Plant EVs may also deliver antifungal molecules to the pathogen. For example, EVs from sunflower seedlings inhibit the growth of *Sclerotinia sclerotiorum* spores (Regente et al., 2017). Plant EVs are also proposed to transport antimicrobial glucosinylates (Rutter and Innes, 2017). Very little is known about EVs produced by filamentous fungi, with data limited to a handful of studies on *Alternaria infectoria* (Silva et al., 2013), *Trichophyton rubrum* (Bitencourt et al., 2018), and *Rhizopus delemar* (Liu et al., 2018). The EVs from the filamentous fungi in these studies are similar to those produced by yeast with respect to morphology, cargo, and recognition by innate immune cells.

To address the lack of knowledge on how EVs contribute to fungal pathogenesis in plants, we have isolated and characterized EVs from the cotton pathogen *Fusarium oxysporum* f. sp. *vasinfectum*. The *F. oxysporum* species complex causes vascular wilt disease in many economically relevant crops and are considered hemibiotrophic pathogens because the infection consists of both a biotrophic and necrotrophic phase (Gordon 2017). The EVs had a similar morphology to those from yeasts and contained protein cargo that was functionally related to those described for human pathogens. EVs from *F. oxysporum* were deep purple in color and were phytotoxic when applied to plant leaves. This supports a role for fungal EVs in the host–pathogen interaction with plants.

## Materials and Methods

### Strains and Growth Conditions

*F. oxysporum* f. sp. *vasinfectum*, Australian isolate VCG01111 isolated from cotton, (gift from Wayne O’Neill, Farming Systems Institute, Department of Primary Industries (DPI), Queensland, Australia), was maintained on half strength potato dextrose agar (1/2 PDA) at 25°C. Liquid cultures were grown in half-strength potato dextrose broth (1/2 PDB) at room temperature with shaking at 90 rpm. *S. cerevisiae* BY4741 were maintained on 1% yeast extract, 2% peptone, 2% dextrose, 2% agar (YPD-agar) at 30°C. Liquid cultures were grown in YPD at 30°C with shaking at 125 rpm.

### Isolation of EVs

EVs were isolated using a procedure modified from Rodrigues et al. (2007). Briefly, *F. oxysporum* f. sp. *vasinfectum* mycelium was cultured in ½ PDB for 72 h at room temperature. Mycelium was removed from the culture medium by filtration through sterile Miracloth. Spores and cell debris were removed from the culture medium by centrifugation at 4,000 x *g* for 15 min in a Heraeus Multifuge X3 centrifuge (Thermo Fisher) followed by 15,000 x *g* for 30 min in an Avanti J-E centrifuge (Beckman Coulter). Supernatant was further centrifuged at 100,000 x *g* using an SW32 rotor in an Optima L-100 XP ultracentrifuge (Beckman Coulter). EV pellets were resuspended in sterile phosphate-buffered saline (PBS). Protein content of EVs was initially assessed using a Qubit fluorimeter (Thermo Fisher) and then separated on SDS-PAGE gels and stained with SYPRO-Ruby (Invitrogen), and protein content was determined according to the manufacturer’s instructions. When needed, EVs were concentrated by pelleting using a TL-100 ultracentrifuge with a TLA100.3 rotor (Beckman Coulter); the supernatant was removed and the pellet resuspended in an appropriate volume to yield the desired concentration.

### Transmission Electron Microscopy

EV samples (5 µl at 0.05 µg/ml protein) were deposited onto carbon-coated 400-mesh copper grids (ProSciTech) that had been glow discharged for 1 min in a K950X turbo evaporator coupled to a K350 glow discharge unit (Quorum Technologies Ltd), incubated for 1 min then washed with ultrapure water. Grids were then stained three times with 4 µl of 2% (v/v) uranyl acetate (Agar Scientific). Excess solution was blotted off and the grids were dried overnight. Images were captured using a JEM 2100 electron microscope (JEOL Ltd) operated at 200 kV.

### Nanoparticle Tracking Analysis

Particle size and number of purified EVs were determined using a Nanosight NS300 with a 405 nm (blue) laser (Malvern Instruments). Samples (0.1 µg/ml) were diluted 1:1,000 with sterile PBS and injected using a syringe pump with a flow rate of 50. Three technical replicates were performed per sample with 1 min videos recorded for analysis. All samples were measured in triplicate and data were analyzed using NTA 3.2 Dev Build 3.2.16 with the auto-analysis settings.

### Proteomic Analysis

Three separate pools of EVs (each pooled from two or three independent biological replicates) with 15 µg of total protein in each, were loaded onto precast NuPAGE^®^ 4–12% Bis-Tris gels in 1x MES SDS running buffer. *Gels* were run at 150 V followed by visualization of proteins with Coomassie stain (Bio-Rad). Gel bands (10) were excised and subjected to in-gel reduction, alkylation, and trypsinization as described in Mathivanan et al. (2012). Briefly, gel bands were reduced with 10 mM DTT (Bio-Rad), alkylated with 25 mM iodoacetamide (Sigma) and digested overnight at 37°C with 150 ng of sequencing grade trypsin (Promega). Tryptic peptides were extracted by 0.1% trifluoroacetic acid in 50% (w/v) acetonitrile and analyzed by LC-MS/MS using LTQ Orbitrap Elite (Thermo Scientific), fitted with nanoflow reversed-phase-HPLC (Ultimate 3000 RSLC, Dionex). The nano-HPLC system was equipped with an Acclaim Pepmap nano-trap column (Dionex—C18, 100 Å, 75 μm × 2 cm) and an Acclaim Pepmap RSLC analytical column (Dionex—C18, 100 Å, 75 μm × 50cm). Typically, for each LC-MS/MS experiment, 3 μl of the peptide mix was loaded onto the enrichment (trap) column at an isocratic flow of 5 μl/min of 3% CH_3_CN containing 0.1% formic acid for 5 min before the enrichment column is switched in-line with the analytical column. The eluents used for the LC were 0.1% v/v formic acid (solvent A) and 100% CH_3_CN/0.1% formic acid v/v. The gradient used was 3% B to 25% B for 23 min, 25% B to 40% B in 2 min, 40% B to 85% B in 2 min, and maintained at 85% B for 2 min before equilibration for 10 min at 3% B prior to the next injection. The Orbitrap Elite MS was operated in the data-dependent mode with a capillary temperature of 250°C, nano ESI spray voltage of +1.9 kv, and S-lens RF value of 60%. All spectra were acquired in positive mode with full scan MS spectra scanning from m/z 300–1,650 in the FT mode at 240,000 resolution after accumulating to a target value of 1.00e6 and maximum accumulation time of 200 ms. Lockmass of 445.12003 m/z was used. For MS/MS, the 20 most intense peptide ions with minimum target value of 2,000 and charge states ≥2 were isolated with isolation window of 1.6 m/z and fragmented by low energy CID with normalized collision energy of 30 and activation Q of 0.25. A whole cell lysate (WCL) sample, prepared by lysing *F. oxysporum* f. sp. *vasinfectum* mycelia using glass beads in a Tissue lyser (Qiagen) and clarifying *via* centrifugation, was also analyzed using the same method for comparison.

### Bioinformatic Analysis of Proteomic Data Set

Mascot Generic File Format (MGF) files were generated using MSConvert with the parameter of peak picking set. X!Tandem VENGEANCE (2015.12.15) was then used to search the MGF files against a target and decoy databases. A predicted protein set for *F. oxysporum* f. sp. *vasinfectum* was downloaded from Ensembl Fungi (FO_Cotton_V1, GCA_000260175) and used to generate the target database. Search parameters used were: fixed modification (carboamidomethylation of cysteine; +57 Da), variable modifications (oxidation of methionine; +16 Da and N-terminal acetylation; +42 Da), three missed tryptic cleavages, 20 ppm peptide mass tolerance, and 0.6 Da fragment ion mass tolerance. Protein identifications were shortlisted to obtain a master list with less than 1% false discovery rate. Proteins that were identified by at least two unique peptides in two of three samples were classified as EV proteins.

Predicted functions of *F. oxysporum* f. sp. *vasinfectum* ORFs were assigned by homology searching using Blast2Go v4.19 with default parameters (Conesa et al., 2005). Functional enrichment analysis was also performed using Blast2Go with Fisher’s exact test. Statistically significant GO terms that were enriched in EV proteins were condensed further to minimize overlap using the reduced GO terms function in Blast2Go.

### Absorbance Spectroscopy of EVs

Absorbance spectroscopy was performed using a SpectraMax m2 spectrophotometer. EVs fractions were loaded into 1 cm pathlength 70 µl UV-Cuvette micro (BRAND, Germany) cuvettes and the absorbance spectra were measured in 10 nm intervals. Data were analyzed using Microsoft Excel.

### Leaf Infiltration Assays

Coker 315 cotton seeds were germinated at 25°C in an Adaptis (Conviron) growth cabinet with 16 h light and 8 h dark for 10 days with 5 seeds per squat pot in standard pine park potting mix. *Nicotiana benthamiana* were grown in an Adaptis (Conviron) growth cabinet with 16 h light and 8 h dark for 4–6 weeks. Infiltrations were performed on the cotton cotyledons or *N. benthamiana* leaves using a protocol modified from Poon et al. (2018) used for infiltration of *Agrobacterium* into the leaves of *N. benthamiana* for transient protein expression. EVs were prepared to a protein concentration of 0.1 µg/ml in 1 ml PBS and infiltrated into the undersides of the cotyledons using a non-luer lock 1 ml syringe (Terumo). Four infiltrations were performed on each leaf. All infiltrations were paired with a PBS control on the second cotyledon of the plant for cotton or on the other half of the leaf for *N. benthamiana*. Cotton cotyledons were also infiltrated with a solution of hyphae and spores that had been removed from the culture supernatant during EV isolation. Hyphae and spores were washed, resuspended in PBS, and diluted until the solution was slightly turbid (OD 600 nm ~ 0.5) and still flowed easily to facilitate infiltration. After infiltration plants were returned to the growth cabinet for 5 days. After 5 days the cotyledons/leaves were removed from the plants and images of lesions on cotyledons were taken using a Nex-7 camera (Sony).

### Optiprep Density Gradient Analysis of EVs

Density gradient ultracentrifugation was performed as described previously with modifications (Kalra et al., 2013). A discontinuous iodixanol gradient consisting of 40% w/v, 20% w/v, 10% w/v, and 5% w/v solutions of iodixanol was prepared by diluting a stock solution of OptiPrep [60% w/v aqueous iodixanol (Sigma)] in 0.25 M sucrose/10 mM Tris, pH 7.5. The crude EVs isolated from the *F. oxysporum* f. sp. *vasinfectum* culture by differential centrifugation coupled with ultracentrifugation, were resuspended in the OptiPrep solution, and overlaid onto the top layer. A control tube consisting 3 ml of each 40%, 20%, 10%, and 5% solutions was also prepared. Both tubes were simultaneously subjected to ultracentrifugation at 100,000 × *g* for 18 h at 4°C (Beckman Coulter: SW-28 rotor). The 12 fractions were collected separately for further analysis. EVs were pelleted by ultracentrifugation at 100,000 × *g* for 1 h at 4°C (Beckman Coulter: TLA-55 rotor). Pellets were then washed with 1 ml of PBS and the supernatant was removed with two successive ultracentrifugations at 100,000 × *g* for 1 h at 4°C (Beckman Coulter: TLA-55 rotor) and resuspended in 30 µl before being stored at -80°C.

## Results

### *F. oxysporum* f. sp. *vasinfectum* Produces EVs

**Figure 1:**
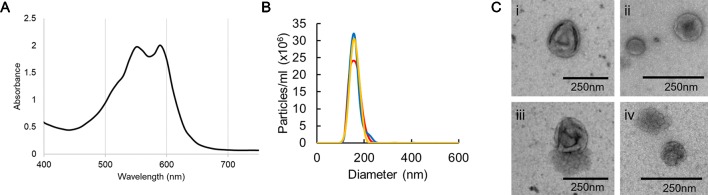
Characterization of EVs isolated from *F. oxysporum* f. sp. *vasinfectum*. EVs were isolated using differential centrifugation followed by ultracentrifugation. **(A)** Absorption spectrum of the *F. oxysporum* f. sp. *vasinfectum* EV preparation had dual maxima at 550 and 590 nm. Data are representative of three independent replicates. **(B)** The number and size distribution of the isolated EVs were determined using nanoparticle tracking analysis. The data from the three technical replicates from one biological sample are representative of all samples used in this study. The average quantity and size of EVs from three independent isolations of EVs from *F. oxysporum* f. sp. *vasinfectum* were 1.0 x 10^12^ ± 2.8 x 10^11^ particles/ml of culture, mean diameter 155.1 nm ± 3.5 nm, mode diameter 150.0 nm ± 5.1 nm. Values are presented ± standard deviation of three biological replicates. **(C)** TEM confirmed that most particles had the characteristic cup like morphology of EVs (i–iii); some particles had a multi-lobed rosette morphology as seen in (iv).

EVs were isolated from the supernatant from *F. oxysporum* f. sp. *vasinfectum* suspension cultures grown in ½ PDB using differential centrifugation and ultracentrifugation. Unexpectedly the EV fraction had a deep purple color and dual absorbance maximum at 550 and 590 nm (**Figure 1A**). Nanoparticle tracking analysis was used to determine the number of particles and the distribution of particle diameters (**Figure 1B**). *F. oxysporum* f. sp. *vasinfectum* produced 1.0 x 10^12^ (st dev 2.8 x 10 ^11^) EVs per mL of culture medium with an average mean particle diameter of 155.1 nm (st dev 3.5 nm) and an average mode of 150.0 nm (st dev 5.1 nm). The average protein concentration of the EV fraction measured by Qubit was 0.05 µg/µl. TEM revealed that the particles had the characteristic cup-like morphology of EVs (**Figure 1C**). In addition to the cup-shaped particles, there were an equivalent number of multi-lobed, rosette-shaped particles observed in the sample.

### *F. oxysporum* f. sp. *vasinfectum* EVs Contain Proteins That Function in a Variety of Cellular Processes

The protein cargo of *F. oxysporum* f. sp. *vasinfectum* EVs was characterized by tryptic digest followed by LC-MS/MS using an LTQ Orbitrap Elite mass spectrometer. The criteria for positive identification of a protein from the mass spectrometry data set were the detection of at least two unique peptides in at least two of three samples. As a result, we identified list of 482 proteins (**Table S1**). The most abundant proteins (**Table 1**) in the EVs included three proteases (FOTG_10608, FOTG_12971, FOTG_06834), two frequency/period clock proteins (FOTG_02405, FOTG_15292), an HSP70 like protein (FOTG_06291), and a polyketide synthase (FOTG_13424) (**Table 1**). An additional polyketide synthase (FOTG_01390) and a fusarubin cluster-esterase (encoded by FOTG_16162) were also identified but at lower abundance.

**Table 1:** The 30 most abundant proteins identified in *F. oxysporum* f. sp. *vasinfectum* EVs.

Protein	Locus	Function	Associated GO terms
**FOTG_08633**	EXM24125	Uncharacterized protein	
**FOTG_10608**	EXM21679	Related to tripeptidyl-peptidase i	F: serine-type endopeptidase activity; P: proteolysis
**FOTG_02405**	EXM33907	Frequency clock protein	C: nucleus; C: cytoplasm; P: regulation of transcription, DNA-templated; P: circadian rhythm
**FOTG_03632**	EXM32021	Glucose-regulated protein	C: Golgi apparatus; C: nuclear membrane; C: luminal surveillance complex; F: ATP binding; F: ATPase activity; F: unfolded protein binding; P: karyogamy involved in conjugation with cellular fusion; P: SRP-dependent cotranslational protein targeting to membrane, translocation; P: response to unfolded protein; P: ER-associated ubiquitin-dependent protein catabolic process; P: posttranslational protein targeting to membrane, translocation; P: *de novo* posttranslational protein folding
**FOTG_15292**	EXM16413	Related to period clock protein frq	C: nucleus; C: cytoplasm; P: regulation of transcription, DNA-templated; P: circadian rhythm
**FOTG_07604**	EXM25889	Uncharacterized protein	
**FOTG_05803**	EXM28573	Protein disulfide-isomerase	C: endoplasmic reticulum; F: protein disulfide isomerase activity; F: FMN binding; F: oxidoreductase activity; P: cell redox homeostasis; P: oxidation–reduction process
**FOTG_06653**	EXM27334	Uncharacterized protein	
**FOTG_03515**	EXM31837	Related to csf1 protein	C: integral component of membrane; P: fermentation
**FOTG_12971**	EXM18981	Cerevisin	F: serine-type endopeptidase activity; P: proteolysis
**FOTG_02417**	EXM33929	Transcription initiation factor tfiid subunit 12	C: SAGA complex; C: transcription factor TFIID complex; C: SLIK (SAGA-like) complex; F: RNA polymerase II activating transcription factor binding; F: chromatin binding; F: translation initiation factor activity; F: TBP-class protein binding; F: protein complex scaffold; F: identical protein binding; F: protein heterodimerization activity; P: regulation of transcription from RNA polymerase II promoter; P: histone acetylation; P: RNA polymerase II transcriptional preinitiation complex assembly; C: chromatin; C: ribosome; P: regulation of translational initiation
**FOTG_15593**	EXM16120	Related to cell wall protein	-
**FOTG_06397**	EXM26950	Related to glu asp-trna amidotransferase subunit a	F: transferase activity; F: carbon–nitrogen ligase activity, with glutamine as amido-N-donor
**FOTG_11173**	EXM21071	Related to oxidoreductase	-
**FOTG_10869**	EXM21345	qi74 protein	F: oxidoreductase activity, acting on CH-OH group of donors; F: flavin adenine dinucleotide binding; P: oxidation–reduction process
**FOTG_08528**	EXM24551	Acetyl- carboxylase	F: acetyl-CoA carboxylase activity; F: biotin carboxylase activity; F: ATP binding; F: metal ion binding; P: fatty acid biosynthetic process; P: pyruvate metabolic process; C: biotin carboxylase complex
**FOTG_13424**	EXM18486	Polyketide synthase	F: oxidoreductase activity; F: transferase activity; F: phosphopantetheine binding; P: oxidation–reduction process
**FOTG_03629**	EXM32018	Probable endonuclease exonuclease phosphatase family protein	F: endonuclease activity; F: exonuclease activity; P: nucleic acid phosphodiester bond hydrolysis
**FOTG_16641**	EXM14982	Probable isoamyl alcohol oxidase	F: oxidoreductase activity, acting on CH-OH group of donors; F: flavin adenine dinucleotide binding; P: oxidation–reduction process
**FOTG_06610**	EXM27272	Elongation factor 3	F: translation elongation factor activity; F: ATP binding; F: ATPase activity; C: ribosome; P: regulation of translational elongation
**FOTG_06834**	EXM26538	Vacuolar protease a	F: aspartic-type endopeptidase activity; P: cellular response to starvation; P: lysosomal microautophagy; P: proteolysis involved in cellular protein catabolic process
**FOTG_03516**	EXM31838	Elongation factor 2	C: integral component of membrane; F: translation elongation factor activity; F: GTPase activity; F: GTP binding; C: ribosome; P: regulation of translational elongation
**FOTG_02422**	EXM33936	atp-citrate synthase subunit 1	C: nucleus; C: cytosol; C: large ribosomal subunit; F: structural constituent of ribosome; F: lyase activity; F: transferase activity, transferring acyl groups, acyl groups converted into alkyl on transfer; F: cofactor binding; P: translation; P: ribosome biogenesis
**FOTG_07981**	EXM24972	Protein mms22	C: nucleus; P: DNA repair; P: replication fork processing
**FOTG_06022**	EXM27593	Calnexin	C: integral component of endoplasmic reticulum membrane; F: calcium ion binding; F: unfolded protein binding; P: protein folding; P: ER-associated ubiquitin-dependent protein catabolic process
**FOTG_03469**	EXM31757	Starch phosphorylase	F: glycogen phosphorylase activity; F: pyridoxal phosphate binding; P: carbohydrate metabolic process
**FOTG_14342**	EXM17503	Related to rf2 protein	F: chitinase activity; F: chitin binding; P: carbohydrate metabolic process; P: chitin catabolic process; P: cell wall macromolecule catabolic process
**FOTG_06291**	EXM28051	hsp70-like protein	C: nucleus; C: cytoplasm; C: integral component of membrane; F: ATP binding; F: unfolded protein binding; P: protein folding; P: SRP-dependent cotranslational protein targeting to membrane, translocation

**Figure 2:**
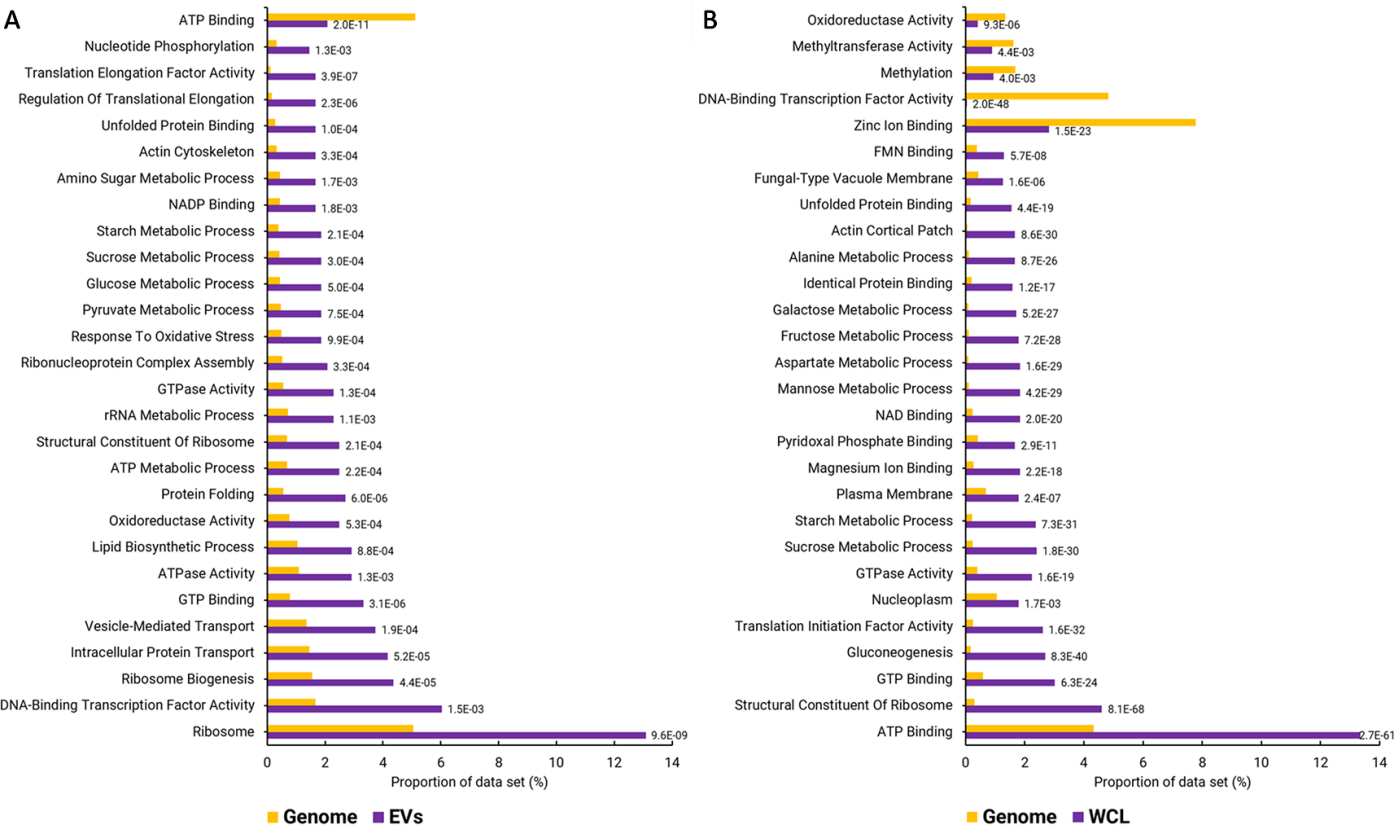
GO terms over- and underrepresented in *F. oxysporum* f. sp. *vasinfectum* EV proteins. **(A)** GO terms that are over- or underrepresented in the set of EV proteins. EV proteins were identified by LC-MS/MS with the peptides aligned to the predicted *F. oxysporum* f. sp. *vasinfectum* proteome. A protein was listed as an EV protein if there were at least two unique peptides from the protein identified in at least two of three EV pools. **(B)** Under- and overrepresented GO terms identified in the list of proteins identified in *F. oxysporum* f. sp. *vasinfectum* whole cell lysate. These lists are the top 30 terms; full lists can be found in **Tables S2** and **S3**. GO terms were assigned to transcripts based on homology searches using Blast2Go. GO term enrichment was also performed using Blast2GO. The number next to each bar is the p-value for the GO term.

Functional enrichment analysis of the EV proteins identified 287 GO terms that were enriched with an adjusted p-value cut-off of 0.05 (**Table S2**). The enriched GO terms were condensed to remove overlapping terms and give a broader picture of the annotated functions, cellular locations, and biological processes that were overrepresented in the *F. oxysporum* EVs (**Figure 2**). A similar enrichment analysis was performed using a proteomic data set from WCL. Basic cellular processes including protein and nucleotide metabolism, lipid biosynthesis, and cell structure were overrepresented EVs. Both the EVs and WCL were enriched for proteins that function in carbohydrate metabolism, the unfolded protein binding, GTP binding, GTPase activity, and ribosomes. Functions such as oxidoreductase activity, oxidative stress response, and vesicle mediated transport were only enriched in EVs. ATP binding was underrepresented in EVs but overrepresented in WCL, whereas DNA-binding transcription factor activity was overrepresented in EVs and underrepresented in WCL.

### *F. oxysporum* f. sp. *vasinfectum* EVs Are Phytotoxic to Plant Leaves

**Figure 3:**
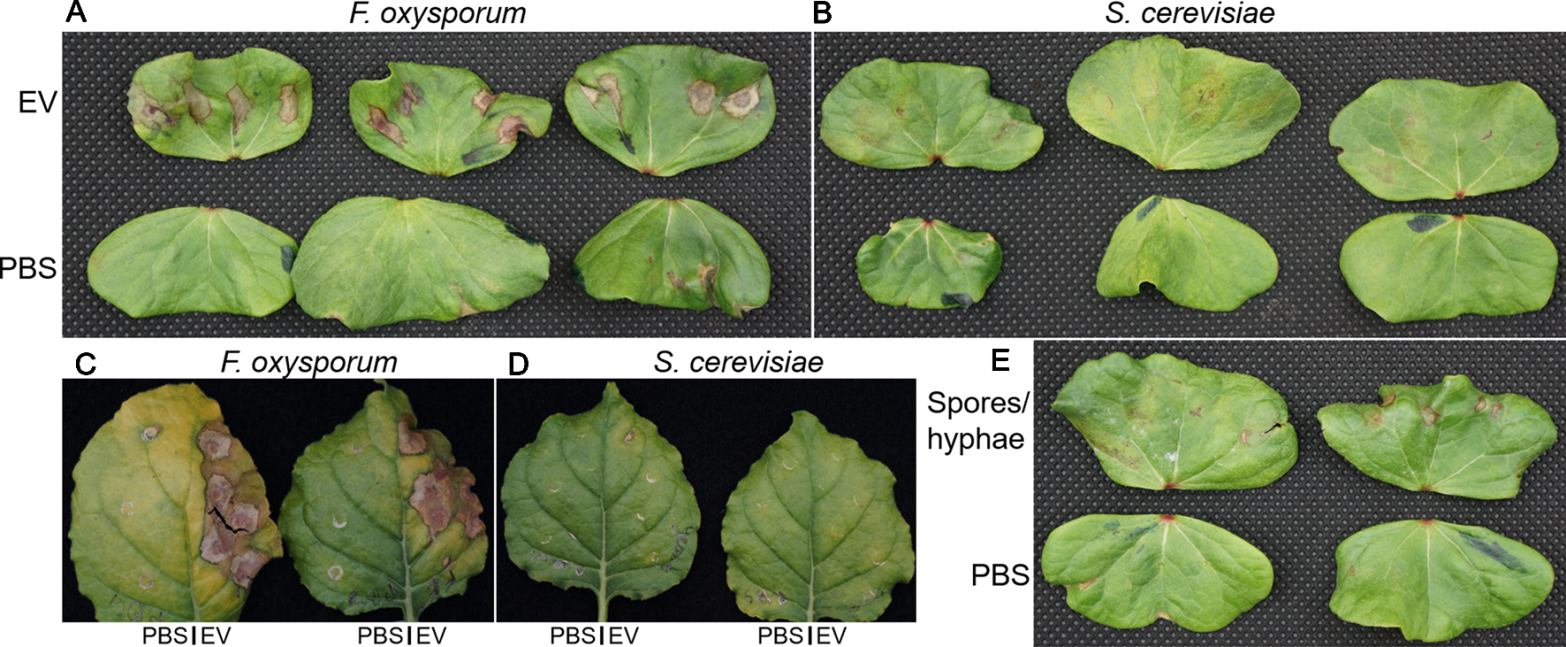
The effect of fungal EVs on cotton cotyledons. EVs were purified from *F. oxysporum* f. sp. *vasinfectum* and *S. cerevisiae* and diluted to a protein concentration of 0.1 µg/ml in PBS. **(A**, **B)** Paired cotyledons were infiltrated at four points with the EV solution and PBS control. After 5 days incubation, the cotyledons were removed from the plants and imaged. Cotyledons infiltrated with EVs from *F. oxysporum* f. sp. *vasinfectum* **(A)** had discolored/brown spots around the sites of infiltration, whereas the cotyledons infiltrated with EVs from *S. cerevisiae* **(B)** only had mild discoloration or no response. Cotyledons infiltrated with PBS had no observable effect (the brown spot on the third cotyledon in the *F. oxysporum* panel **(A)** is due to a pinch point as a result of a fold in the leaf and not infiltration). **(C**, **D)** *N. benthamiana* leaves were infiltrated with EVs at multiple points on the right half and PBS on the left. After 5 days the leaves were removed from the plants and imaged. Leaf halves infiltrated with EVs from *F. oxysporum* f. sp. *vasinfectum* **(C)** showed substantial discoloration around the sites of infiltration, whereas the half infiltrated with PBS had no discoloration. Leaf halves infiltrated with EVs from *S. cerevisiae* **(D)** appeared similar to the corresponding PBS infiltrated leave halves. Infiltration of cotton cotyledons with resuspended *F. oxysporum* f. sp. *vasinfectum* spores and hyphae **(E)** led to the formation of very small discolored spots at the sites of infiltration, but they were much smaller than those observed with EVs. Images are representative of three independent experiments.

To determine whether EVs produced by *F. oxysporum* f. sp. *vasinfectum* had any effect on plant tissue, EVs were infiltrated into the underside of the cotyledons of cotton seedlings or young leaves of *N. benthamiana*. After 5 days the *F. oxysporum* f. sp. *vasinfectum* EVs induced the formation of a discolored area on the cotyledon or leaf corresponding to the infiltration area, which is indicative of phytotoxicity (**Figure 3A**). In contrast, there was no discoloration in the infiltration areas of any of the cotyledons or leaves that were infiltrated with sterile PBS demonstrating that the infiltration procedure itself was not responsible for the discolored region. Infiltration of resuspended spores and hyphae led to the formation of discolored spots that were much smaller than those observed for the EVs. To determine whether the toxicity observed in the cotyledon was merely a response to foreign material or if it was specific to components of the *F. oxysporum* f. sp. *vasinfectum* EVs, the infiltration experiment was repeated with EVs isolated from the non-pathogenic fungus *S. cerevisiae*. No discoloration was observed in the regions infiltrated with *S. cerevisiae* EVs; indeed it was difficult to discern between the *S. cerevisiae* EV infiltrated and the paired PBS infiltrated cotyledons (**Figure 3B**). As *F. oxysporum* f. sp. *vasinfectum* is a specific pathogen of cotton, the infiltration experiment was repeated on *N. benthamiana* to determine if the phytotoxic effect was host specific. Infiltration of EVs from *F. oxysporum* f. sp. *vasinfectum* was also phytotoxic on *N. benthamiana* leaves (**Figure 3C**), indicating that the phytotoxic effect is not host specific. Consistent with observations in cotton, *S. cerevisiae* EVs did not elicit a response in *N. benthamiana* leaves (**Figure 3D**). Infiltration of cotton cotyledons with resuspended spores and hyphae led to a much-subdued phytotoxic effect (**Figure 3E**), indicating that it is not just a response to fungal material that causes the phytotoxic response in plants but something specific to EVs.

**Figure 4:**
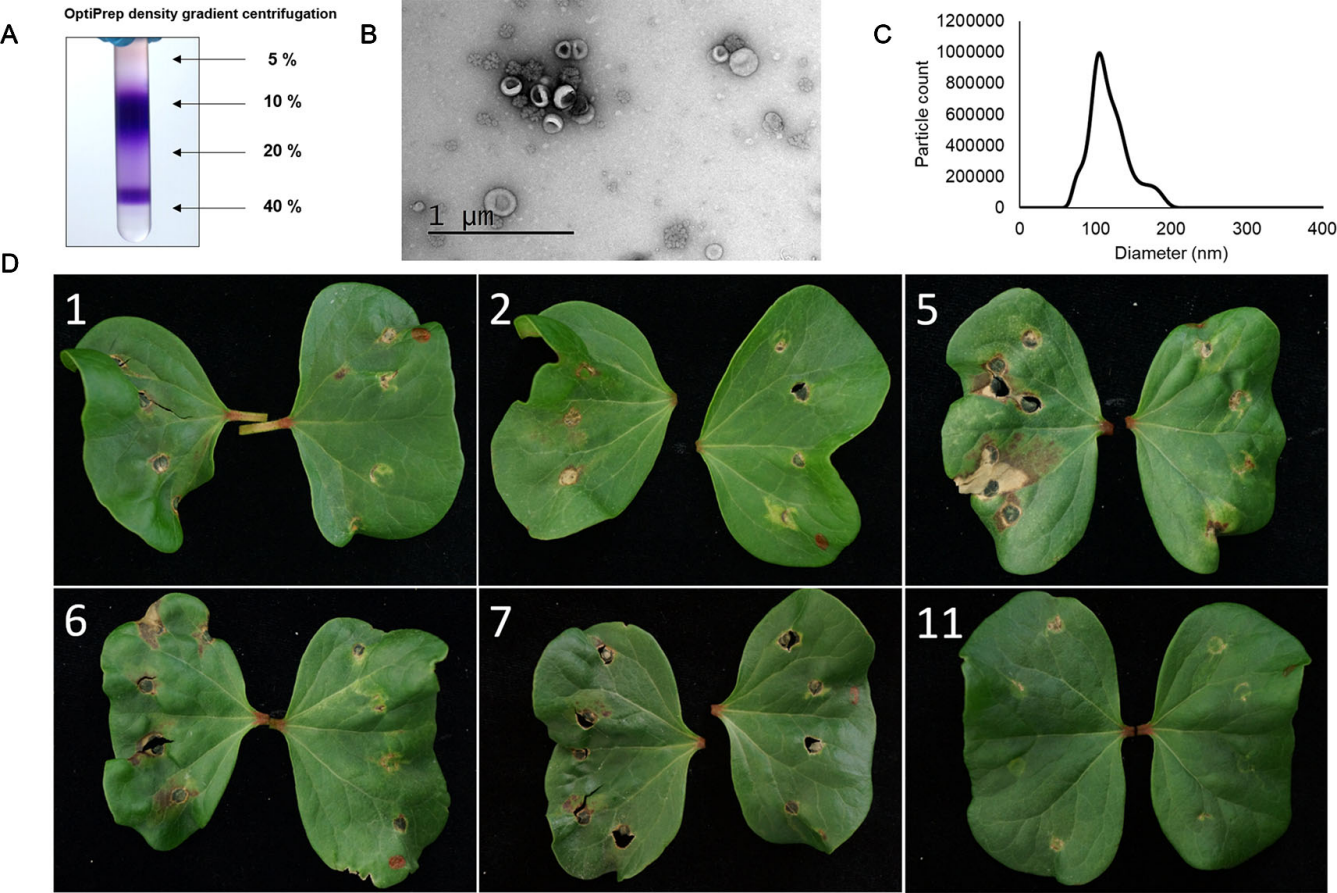
The optiprep fractions containing both EVs and the purple pigment are responsible for the hypersensitive response induced by the crude EV fraction in plants. **(A)** Optiprep gradient centrifugation of the crude EV fraction revealed that the purple pigment concentrates in two regions, one centered at 10% and a second close to 40% iodixanol. The purple pigment was concentrated in fractions 5–7. **(B)** Representative TEM of fractions 5–7. Particles with characteristic cup-like morphology of EVs were detected in these fractions as well as some rosette-shaped particles. **(C)** Representative NTA of fractions 5–7. The mode particle diameter was 104.5 nm; no particles were detected below 46.5 nm or above 226.5 nm. **(D)** Application of select fractions (numbered in white) to the cotyledons of a cotton plant. Fractions (left cotyledon) were all paired with a PBS control (right cotyledon). Fraction 5 induced the strongest response with fractions 6 and 7 inducing a lesser response. Fractions 1, 2, and 11 did not induce a response beyond that observed for the PBS control. The brown spots and holes on the control cotyledons are due to wounding of the leaf during infiltration.

As there are several different molecules produced by fungi that can induce a cell death response in plants further purification of the *F. oxysporum* f. sp. *vasinfectum* EVs by Optiprep density gradient centrifugation was performed. The purple pigment separated into two sections of the Optiprep gradient. The first section spanned fractions 4–7 and a second more condensed section in fraction 10 (**Figure 4A**). After removal of the iodixanol, a selection of these fractions were infiltrated into cotton cotyledons. Fractions 5, 6, and 7 induced the strongest response and also contained an enrichment of EVs (**Figures 4B**–**D**). Co-purification of the pigment with the EVs on the optiprep gradient supports the notion that the pigment is packaged in the EVs. The phytotoxicity of these fractions on cotton plants indicated that EVs and/or the pigment are responsible for initiating the response.

## Discussion

We have isolated EVs from the plant pathogen *F. oxysporum* f. sp. *vasinfectum*, characterized their protein cargo, and discovered that they have a phytotoxic effect when applied to the leaves of cotton and *N. benthamiana*. A unique feature of the EVs isolated from *F. oxysporum* f. sp. *vasinfectum* was their deep purple color, which has not been reported for EVs from any other species.

There are no marker proteins for fungal EVs so confirmation that the particles we had isolated *via* ultracentrifugation were indeed EVs depended on physical characterization. TEM confirmed that the EVs isolated from *F. oxysporum* f. sp. *vasinfectum* had the characteristic morphology of EVs from other organisms. NTA confirmed that the population of EVs had a similar size distribution to those reported for *C. albicans* and *C*. *neoformans* that also used NTA for size analysis (Bielska and May, 2019). Larger vesicles (diameter greater than 450 nm) have been detected in *C. neoformans, C. albicans*, and *Malassezia sympodialis* EV preparations (Bielska and May, 2019) but were not detected in this analysis. The rosette-shaped particles have been described previously in an isolation of EVs from *S. cerevisiae*, but their origin and identity remain unknown (Giardina et al., 2014).

Proteomic characterization of EVs from *F. oxysporum* f. sp. *vasinfectum* was conducted to elucidate potential functions of these particles in the host–pathogen interaction. Proteomic analysis *F. oxysporum* f. sp. *vasinfectum* EVs identified 482 proteins, which is greater than the 205 (or fewer) proteins identified in the early studies on yeast EVs (Rodrigues et al., 2008; Vallejo et al., 2012; Gil-Bona et al., 2014; Wolf et al., 2014; Vargas et al., 2015; Wolf et al., 2015) and lower than the 600 (or greater) reported from *S. cerevisiae* and *C. albicans* EVs obtained using similar methods of isolation and proteomic analysis (Zhao et al., 2019; unpublished data from the Anderson lab). This may have arisen from the lower yield of EVs from *F. oxysporum* f. sp. *vasinfectum* compared to yeast cultures. Functional enrichment analysis of the *F. oxysporum* f. sp. *vasinfectum* EV cargo proteins mostly identified basic cellular metabolic and biosynthetic processes. In addition, there were many proteins with unknown function. Yeast EVs are also enriched for proteins that function in these basal cellular processes as well as proteins that function in pathogenesis, stress responses, and cell wall dynamics (Bleackley et al., 2019). The latter protein groups were not identified in the *F. oxysporum* f. sp. *vasinfectum* EVs but this is probably due to the relatively poor annotation of the genome of *F. oxysporum* f. sp. *vasinfectum*. It is likely that a subset of the uncharacterized proteins, as well as proteins that have been incorrectly annotated based on sequence homology, have a function in *F. oxysporum* f. sp. *vasinfectum* pathogenesis.

An Hsp70 like protein was also found in *F. oxysporum* f. sp. *vasinfectum* EVs. Hsp70 was one of the few identified in all previously published proteomic data sets for *C. albicans* and *C. neoformans* EVs (reviewed in Bleackley et al., 2019) and is also found in mammalian exosomes (Lancaster and Febbraio, 2005). Hsp70 proteins are molecular chaperones that assist in protein folding and stability (Mayer and Bukau, 2005) that have been proposed as molecules that communicate intercellular stresses through packaging into EVs (De Maio, 2011). HSP70 may prove to be a valuable marker for fungal EVs. Marker proteins in mammalian EVs facilitate analysis of EV purity, that is, from potential contamination from organelle fragments (Lötvall et al., 2014), and the lack of markers for fungal EVs means that this is not currently possible.

One of the most striking observations in this study was the deep purple color of the EVs isolated from *F. oxysporum* f. sp. *vasinfectum*. The pigment not only co-purified with the EVs during ultracentrifugation but also co-purified with the EVs during the Optiprep gradient analysis. Packaging of pigments into EVs could represent a conserved secretion mechanism across fungi because melanin, a pigment that has a role in the virulence of *C. neoformans*, is also packaged into a subset of EVs (Rodrigues et al., 2008). Proteomic analysis of EVs isolated from *A. infectoria*, which also contain melanin, identified a polyketide synthase involved in biosynthesis of melanin and other pigments as EV cargo (Silva et al., 2013). *Fusarium* spp., like most fungi, produce a wide variety of secondary metabolites, which include mycotoxins that negatively impact agricultural production as well as molecules that have served as a starting point for development of pharmaceuticals (Brown et al., 2012). The source of the purple color was initially suspected to be a polyketide secondary metabolite similar to purpurfusarin, which was identified from a *F. graminearum* strain over expressing *PGL1* (Frandsen et al., 2016). Purple pigments have since been isolated from *F. oxysporum* cultures grown on defined minimal dextrose broth (Lebeau et al., 2019). The absorbance spectra of an ethanol extract of lyophilized culture medium was consistent with the dual maxima at 550 and 590 nm of the EVs. These purple pigments were further refined and had typical UV-Vis absorbance profiles for napthoquinones, but specific peaks in the UV spectra indicated that they are not purpurfusarin (Frandsen et al., 2016) or 8-*O*-methyl anhydrofusarubin (Studt et al., 2012), which are known purple pigments from *Fusarium*. Bikaverin, a polyketide with antibiotic properties (Limón et al., 2010), was also detected in the extracts containing the pigments (Lebeau et al., 2019); it follows that perhaps bikaverin is also packaged into EVs. Analysis of the small molecule content of EVs is required to further enhance understanding of potential role of EVs in the secretion of polyketides and other fungal metabolites. Identification of the two polyketide synthases and one fusarubin cluster-esterase as EV cargo in this study could point towards EVs as a site of biosynthesis for pigments and/or toxins.

We speculated that *F. oxysporum* f. sp. *vasinfectum* EVs had a role in the fungal–plant interaction because the role of EVs in bacterial infections is well described in mammalian systems (Pathirana and Kaparakis-Liaskos, 2016). Furthermore, pathogenic yeasts such as *C. albicans, C. neoformans*, and *Paracoccidioides brasiliensis* are able to activate innate immune cells such as macrophages (Oliveira et al., 2010) and dendritic cells (Vargas et al., 2015; Wolf et al., 2015). EVs are also a contributing factor to the hypervirulence phenotype of a strain of *C. gattii* (Bielska et al., 2018). This led to the hypothesis that EVs would play a role in fungal pathogenesis in plants. EVs from *F. oxysporum* f. sp. *vasinfectum* that were infiltrated into cotyledons of cotton or leaves of *N. benthamiana* were phytotoxic. This toxicity was not merely a response to fungal cell wall polysaccharides or lipids as there was no phytotoxic response to EVs from *S. cerevisiae* or to intact fungal spores and hyphae from *F. oxysporum* f. sp. *vasinfectum*. EVs as the source of the phytotoxicity was further supported by the observation that after further fractionation of the initial EV prep using Optiprep gradient centrifugation, the EV enriched fractions retained phytotoxic activity whereas fractions that did not contain EVs did not. Although the leaf is not the normal route of infection for *F. oxysporum* f. sp. *vasinfectum*, the effect of EVs on leaf tissue demonstrates that the EVs do carry phytotoxic compounds. Furthermore, a similar phytotoxic effect was observed the *F. oxysporum* f. sp. *vasinfectum* EVs were infiltrated into *N. benthamiana*, a plant that is not a host for *F. oxysporum* f. sp. *vasinfectum*, demonstrates that the EVs contain a phytotoxic compound and it is not a host specific virulence factor that is eliciting this response. This could be related to the pigments and/or other polyketides that are associated with the EVs as these molecules can have a range of detrimental effects on plant cells (Möbius and Hertweck, 2009). Alternatively, EV proteins could be the cause of the phytotoxicity. Several proteases, a family of proteins that contribute to pathogenesis of various plant pathogens (Olivieri et al., 2002; Yike, 2011; Lowe et al., 2015), were packaged into EVs and could play a key role in contributing to the damage caused by the EVs. The phytotoxic effect of EVs during an infection is likely to be less extreme that the one observed here due to the amount of EVs used in these experiments compared to what would be present during an infection. EVs were isolated from a 1 L culture and resuspended in approximately 2 ml of PBS prior to infiltration and exceeds what would be expected to be produced by the fungus at any one point during an infection. However, a fungus would continuously be producing EVs during an infection so the cumulative exposure to EVs would increase over time. The high concentration, single dose of EVs was used to ensure that an effect was observed upon infiltration. Future investigation on the role of EVs in an infection will need to investigate physiologically relevant EV concentrations and use more sensitive techniques to measure the response in the plant.

In summary, *F. oxysporum* f. sp. *vasinfectum* produces EVs similar in morphology and size to EVs from other fungal species. These EVs carry protein cargo and pigments that may have a role in pathogenesis. Preliminary data demonstrated that EVs from *F. oxysporum* f. sp. *vasinfectum* are phytotoxic to leaves of both its host plant cotton and the unrelated plant *N. benthamiana*. This may be a response turned on by the plant in recognition of the fungal EVs to limit the spread of infection and may explain why *F. oxysporum* f. sp. *vasinfectum* enters the plant through the roots instead of the leaves. Alternatively, EV release could be downregulated during the biotrophic phase of the infection and then turned on when the fungus switches to necrotrophy. Further characterization of EVs from *F. oxysporum* f. sp. *vasinfectum* and other agricultural fungal pathogens will improve our understanding of the plant–pathogen interaction and may ultimately lead to identification of new targets to enhance disease protection in crop plants.

## Data Availability Statement

All datasets for this study are included in the article/**Supplementary Material**.

## Author Contributions

MB performed infiltration assays, analyzed proteomic data, and wrote the manuscript. MS performed EV isolations and characterization, prepared samples for mass spec, and performed infiltration assays. DG-C performed EV isolations and characterization, prepared samples for mass spec. JM aided in design of infiltration assays and collected images from infiltration assays. RL annotated the proteome and analyzed proteomic data. MP analyzed proteomic data. KZ isolated yeast EVs. C-SA performed proteomic data collection. SM conceived the project, oversaw the experimental work and data analysis. MA conceived the project, oversaw the experimental work and data analysis, and revised the manuscript.

## Funding

This work was funded by Australian Research Council grants to MA (DP160100309) and SM (DP170102312 and DE150101777). JM is a Commonwealth Scientific and Industrial Research Organisation Science and Industry Endowment Fund STEM Fellow.

## Conflict of Interest

The authors declare that the research was conducted in the absence of any commercial or financial relationships that could be construed as a potential conflict of interest.

## Supplementary Material

The Supplementary Material for this article can be found online at: https://www.frontiersin.org/articles/10.3389/fpls.2019.01610/full#supplementary-material

Table S1 Full list of proteins in EVs.

Table S2 EV enriched GO terms.

Table S3 WCL enriched GO terms.
